# Finding the needle in a haystack: Mapping antifungal drug resistance in fungal pathogen by genomic approaches

**DOI:** 10.1371/journal.ppat.1007478

**Published:** 2019-01-31

**Authors:** Dominique Sanglard

**Affiliations:** Institute of Microbiology, Lausanne University Hospital and University of Lausanne, Lausanne, Switzerland; Geisel School of Medicine at Dartmouth, UNITED STATES

## Fungi strike back when attacked

Fungi are ubiquitous on earth and are essential for the maintenance of the global ecological equilibrium. Despite providing benefits to living organisms, they can also target specific hosts and inflict damage. These fungal pathogens are known to affect, for example, plants and mammals and thus reduce crop production necessary to sustain food supply and cause mortality in humans and animals. Designing defenses against these fungi is essential for the control of food resources and human health. As far as fungal pathogens are concerned, the principal option has been the use of antifungal agents, also called fungicides when they are used in the environment.

**Fig 1:**
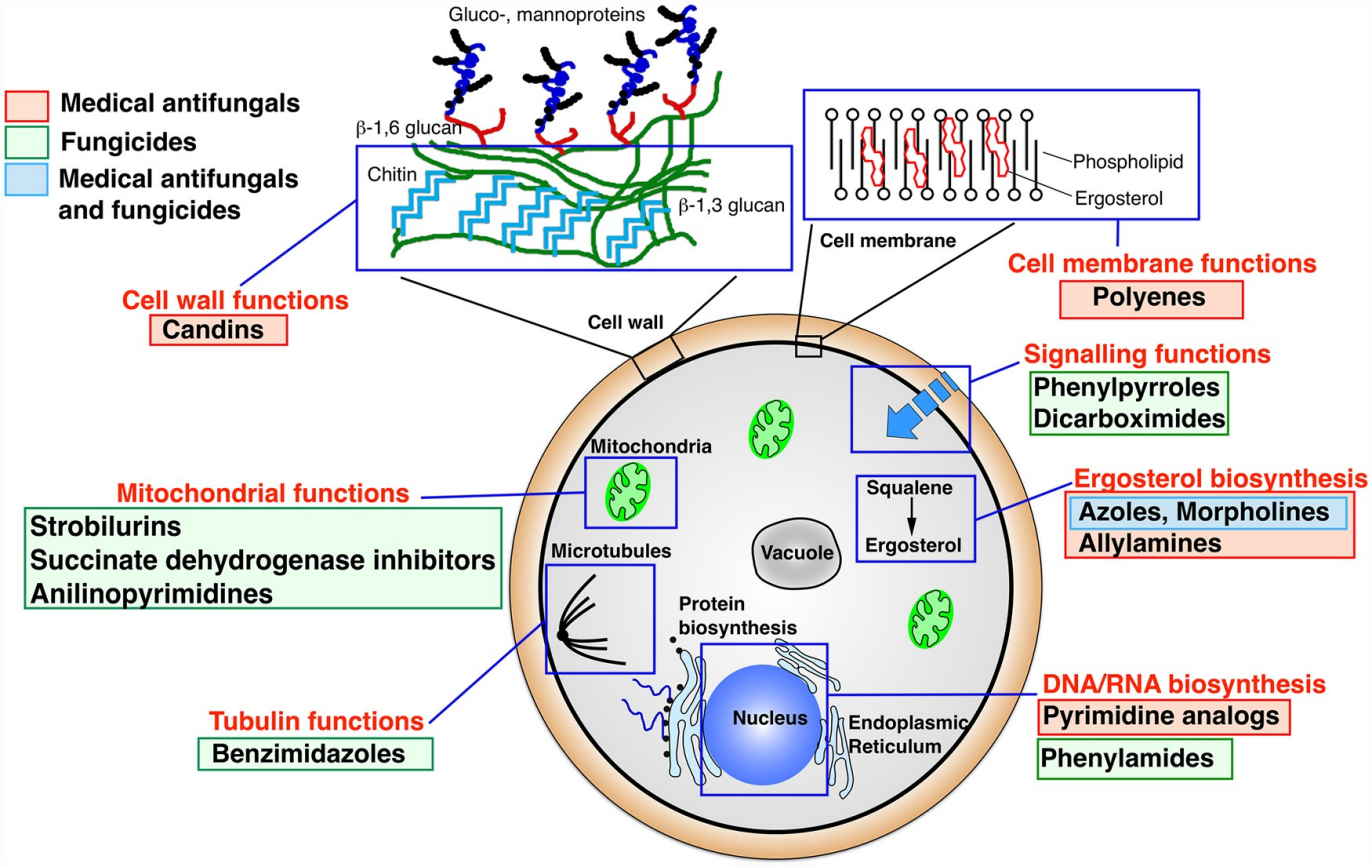
Cellular targets of principal antifungals used in medicine and in agriculture. The cited antifungals and fungicides have the following mode of action: polyenes bind ergosterol; phenylpyrroles and dicarboximides inhibit MAP/histidine-kinases; azoles and morpholines target 14α-lanosterol demethylase and both sterol Δ14-reductase and Δ8-Δ7-isomerase, respectively (both chemical classes are used in medicine and in the environment); allylamines target squalene epoxidase; pyrimidine analogues interfere with nucleic acid biosynthesis whereas phenylamides target RNA polymerase I; benzimidazoles bind to b-tubulin; strobilurins and succinate dehydrogenase inhibitors inhibit both the electron transfer chain of mitochondrial respiration by inhibiting complex III and complex II, respectively; anilinopyrimidines target mitochondrial signaling pathways; candins target the biosynthesis of β-1,3 glucans. MAP, Mitogen-activated protein.

Commercial antifungal agents and fungicides belong to several classes and affect several cellular functions (Fig 1) [1]. Fungicides outnumber medical antifungal agents by the number of chemical classes and by the number of licensed drugs [1]. Exposing plant and/or human pathogens to these agents results in a substantial fungal growth deficiency and eventually clearance of the pathogens in the infected sites or infected host. However, the same exposure imposes strong selective pressures for the development of resistance, which is ineluctably acquired over short- and long-term periods. Fungicides that are spread in the environment can also cause the selection of resistance in ubiquitous fungi that are also human pathogens. The transmission of environmentally acquired resistance to human is of concern because it reduces the limited number of possible treatments to a few alternatives.

It is important to resolve resistance mechanisms because they may highlight not only novel biology but also may reveal novel strategies to counteract and combat resistance. Resistance mechanisms can be grouped in at least three general principles that include (i) decrease of effective intracellular drug concentration, (ii) alterations of the drug target, and (iii) compensatory mechanisms which decrease drug toxicity [2]. I will summarize here approaches that led to the understanding of antifungal resistance in plant and human fungal pathogens with a special focus on genome-wide studies.

## Reverse and forward genetics to understand drug resistance

Genome-wide studies performed on the yeast *Saccharomyces cerevisiae* have been crucial to decipher the mode of action of drugs and associated resistance mechanisms [3]. These studies are based on reverse genetics with the use of mutant collections and/or overexpression collections that can be scaled to high throughput screenings [4]. Genetic screenings can reveal not only the principal target of antifungal agents but also identify off-targets which constitute genetic networks that are affected by drug exposure [5]. Several examples of such genetic screens have been reported in human fungal pathogens. In *Candida albicans*, homozygous and heterozygous mutant collections (a total of 3,030 mutants) were used to identify gene networks that contribute to resistance to candins. In the same species, overexpression of all transcription factors of a specific family (zinc cluster family) helped to identify novel mediators of azole resistance [6]. In *C*. *glabrata*, a collection of deletion mutants (619) was used to identify genes critical for azole and candin stress [7]. In *Cryptococcus neoformans*, a collection of transcription factor mutants (322) explored gene networks mediating resistance to azoles, polyenes, and 5-fluorocytosine [8].

Forward genetics and the systematic generation of drug-resistant mutants can also be used to discover the mode of action of drugs. The identification of the mutation(s) responsible for the drug resistance phenotype can be addressed by classical genetic analysis of the mutants. This requires, however, several lengthy processes, including, for example, back-crosses with tester strains and/or complementation tests with whole-genome libraries [9]. A classic example in early studies is the discovery of the targets of rapamycin using mutants generated in *S*. *cerevisiae* [10].

This classical forward genetic approach can now be greatly facilitated by whole-genome sequence analysis. With the technical advances of DNA sequencing technologies, whole genomes can now be sequenced with high efficiency and at low cost. As early as 2008, the use of a whole-genome approach (by pyrosequencing) addressed the occurrence of spontaneous mutations in *S*. *cerevisiae* [11]. This study was followed in 2010 by another genome-scale study (using pyrosequencing) in *S*. *cerevisiae* that identified gain of function alleles of peroxiredoxin (*TSA1*) which conferred resistance to oxidative stress [12]. Elucidation of drug resistance mechanisms has recently progressed by the use of hypermutator strains in *S*. *cerevisiae* lacking a functional *MSH2* gene [13]. This gene participates in DNA mismatch repair, and when its activity is decreased either by mutations or gene inactivation, it induces an increased rate of spontaneous mutations or drug-induced mutations [14]. The study of Ojini and colleagues [13] exploited this property to generate mutants resistant to several drugs including canavanine, camptothecin, and several anticancer drugs. Systematic genome sequencing of drug-resistant mutants revealed mutations in genes critical for drug resistance, e.g., *CAN1* for canavanine and *TOP1* for camptothecin [13]. The use of hypermutator *S*. *cerevisiae* strains with an *MSH2* defect followed by whole-genome sequencing was recently used to identify the gene (*ERG6*) targeted by the natural antifungal compound tomatidine [15]. These drug resistance mapping studies underline the great potential of whole-genome sequencing. This specific use of genome sequencing is, however, only a small fraction of the vast majority of other genome sequencing studies performed with fungi that were aimed to resolve population genetic structures in *S*. *cerevisiae* [16] or even *C*. *albicans* [17].

## Genome-wide studies to understand drug resistance in fungal pathogens

Drug-resistant fungal pathogens originating from the environment or from the clinic constitute an immense resource for understanding antifungal drug resistance mechanisms. Whole-genome sequence data and downstream analysis give unique opportunities to identify different types of mutations, including single nucleotide polymorphisms (SNPs), insertions, deletions, or structural variations (gene or chromosome copy variations including aneuploidies) involved in drug resistance. At least two approaches can deliver suitable candidate nucleotide positions in genome-wide analysis. The first is inspired by human genetics and makes use of genome-wide association studies (GWAS). Quantitative trait loci (QTLs) analysis is also aimed at resolving the relationships between phenotypes and genotypes but not at the nucleotide resolution level of GWAS. The basic principle of GWAS is to screen diverse populations for phenotypic differences between individuals. Although many GWAS performed on fungal plant pathogens have focused on resistance to host factors [18], a few have addressed the identification of drug resistance genes with this approach. A landmark study was published by Mohd-Assaad and colleagues [19], in which azole resistance was investigated in *Rhynchosporium commune*, the causal agent of the barley scald disease. The study used whole-genome data from a set of 120 isolates and identified *CYP51A* mutations (the target of azoles) and several other loci (*YVC1*, a calcium channel, a transcription activator, and a saccharopine dehydrogenase) that also contributed to azole resistance. Another GWAS [20] on *Fusarium graminearum* (a fungus causing *Fusarium* head blight) mapped several phenotypes, including azole resistance. It is important to note that the genome data obtained here were from 119 isolates through restriction site-associated DNA sequencing (RADseq), which can provide a large number of SNP markers but at a lower resolution than whole-genome sequencing using high-throughput methods. The study identified 51 genes associated with propiconazole sensitivity in wild-type strains phenotyped under natural field conditions. This provided useful insights into non-*CYP51A* mediated resistance mediators. It is likely that other GWAS will emerge in the future; even if GWAS show great potential, they also have significant limitations. The structure of the investigated populations is of critical importance. For example, GWAS are more likely to succeed if (i) the investigated population is undergoing sexual recombination, (ii) the number of isolates originates from diverse conditions, and (iii) the number of investigated isolates is above a threshold of approximately 100 isolates [21]. Fungal species, including *C*. *albicans* [17] and *C*. *glabrata* [22] or filamentous fungi such as *Aspergillus fumigatus* [23], have marginal or absent sexual reproduction. Therefore, the power of GWAS is predicted to be limited in these clonal species. Despite this limitation, GWAS was successfully undertaken in a study of 387 *C*. *neoformans var*. *grubii* genomes and identified 10 loci associated with virulence capacity [24]. The study also investigated melanization in these isolates and identified a loss of function in *BZP4*, which is a transcription factor required to produce melanin, a molecule necessary for maintaining *Cryptococcus* virulence [24]. Another study performed on only 20 *C*. *albicans* isolates linked host-associated phenotypic traits with individual loci in the genome. A nonfilamentous isolate exhibited a nonsense mutation in *EFG1*, a gene involved in yeast-to-hyphae transition [25].

## Finding the needle in a haystack

Genome-wide studies aimed to resolve drug resistance mechanisms on human pathogens are using in their majority genome comparisons between investigated isolates. The included isolates involve sometimes isogenic strains or strains with a high degree of relatedness. Studies enumerated in Table 1 give an overview of genome-based approaches available so far to understand drug resistance in fungal pathogens of clinical importance. A first aspect to consider is whether genomic data were obtained via de novo genome assemblies or through read alignments to reference genomes. De novo assemblies can capture genomic rearrangements and resolve individual gene copy number variations that could mediate drug resistance, which is beneficial for these purposes as compared to read alignments to reference genomes. On the other hand, whole chromosome or segmental aneuploidies can be the cause of drug resistance as reported in *C*. *albicans* [26] and *C*. *neoformans* [27]. These changes can be readily detected using read alignment strategies. Most of the genome data obtained in the studies from Table 1 are the results of read alignments, with the exception of specific *C*. *auris* and *C*. *glabrata* genome comparisons [28,29].

**Table 1:** Genome-wide studies from antifungal drug-resistant isolates.

Fungal species (ploidity)	Number of investigated isolates	Genome sequencing technology/Bioproject/SRA/ENA	Major findings	Antifungal class	Genetic validation of identified SNPs	Reference
*C*. *albicans* (2n)	43^a^	Base paired-end Illumina sequencing/ PRJNA257929	LOHs associated with drug resistance *ERG11* and *TAC1* SNPs	azoles	no	[30]
*C*. *albicans* (2n)	1	Illumina HiSeq platform/ PRJNA194436	*ERG2* loss of function (retrotransposon insertion) *ERG6* loss of function (SNP)	Amphotericin B	yes	[37]
*C*. *albicans* (2n)	6	454 Sequencing System^b^	*FKS1* SNP *ERG3* SNPs	Echninocandins Azoles	no	[38]
*C*. *albicans* (2n)	5	MiSeq sequencing (Illumina)/NA	*TAC1*, *MRR1*, *ERG11*, *ERG3*, *UPC2* SNPs	Azoles	no	[39]
*C*. *auris* (1n)	25	Illumina HiSeq 2500/ MinION/PRJNA392455	*ERG11* SNP *FKS1* SNP *FUR1* SNP	Azoles Echinocandins 5-FC	no	[40]
*C*. *auris* (1n)	47	Pacbio/Illumina HiSeq/ PRJNA328792	*ERG11* SNPs	Azoles	no	[28]
*C*. *auris* (1n)	52	Illumina HiSeq 2500/NA	utg5_821828 (*FLO8*) SNP	Amphotericin B	no	[41]
*C*. *glabrata* (1n)	2	Illumina Genome Analyzer II platform/ SRA047280.2	*FKS2* SNP *CDC6* SNPs	Echinocandins	yes	[42]
*C*. *glabrata* (1n)	3	454 Sequencing System^b^	*FKS1*, *FKS2* SNPs *CgPDR1* SNPs	Echninocandins Azoles	no	[38]
*C*. *glabrata* (1n)	12	Illumina NextSeq 500 platform/PRJNA310957	*FKS1* and *FKS2* SNPs *CgPDR1* SNPs	Echinocandins Azoles	no	[43]
*C*. *glabrata* (1n)	1	Ion Torrent/PRJEB15305	*CgPDR1* SNP	Azoles	no	[44]
*C*. *glabrata* (1n)	2	PacBio/ PRJNA374542	*CgPDR1* SNP	Azoles	yes	[29]
*C*. *parapsilosis* (2n)	2	Ion Torrent/PRJNA361149	*ERG3* SNP	Azoles	yes	[45]
*C*. *parapsilosis* (2n)	2	Illumina HiSeq 4000/ SRP077071	*ERG3* SNP	Azoles	yes	[32]
*C*. *lusitaniae* (1n)	20	Illumina NextSeq500/PRJNA433226	*MRR1* SNPs	Azoles	yes	[33]
*C*. *tropicalis* (2n)	1	Illumina HiSeq platform/ PRJNA194436	*ERG3* loss of function (SNP) *ERG11* loss of function (170 bp deletion)	Amphotericin B	yes	[37]
*A*. *fumigatus* (1n)	24	Illumina HiSeq 2500/PRJEB8623	*CYP51A* SNPs	Azoles	no	[46]
*A*. *fumigatus* (1n)	13	Illumina NextSeq 500 platform/NA	*CYP51A* SNPs	Azoles	no	[47]
*A*. *fumigatus* (1n)	2	Illumina Genome Analyzer II/ ERP001097	*HapE* SNP	Azoles	yes	[48]
*A*. *fumigatus* (1n)	37^c^	Illumina Genome Analyzer II/ PRJNA237785	*CYP51A* SNPs *ERG25* SNP *GanA* SNP	Azoles	no	[31]
*Cryptococcus deuterogattii* (1n)	4^c^	NA/PRJNA387047	*FRR1* SNPs	FK506/rapamycin	yes	[49]

^a^ Paired isolates from 11 patients.

^b^ Six drug resistance genes were amplified from six isolates and sequenced in a single run.

^c^ Experimental in vitro adaptation.

**Abbreviations**: *CDC6*: cell division cycle 6; *CgPDR1*: *Candida glabrata* Pleiotropic drug resistance 1; *CYP51A*: 14α-lanosterol demethylase; ENA,; *ERG2*: C-8 sterol isomerase; *ERG3*: C-5 sterol desaturase; *ERG6*: Delta(24)-sterol C-methyltransferase; *ERG11*: 14α-lanosterol demethylase; *ERG25*: C-4 methyl sterol oxidase; *FKS1*: 1,3-beta-D-glucan synthase 1; *FKS2*: 1,3-beta-D-glucan synthase 2; *FLO8*: Floculation gene 8; *FRR1*: FK506-binding protein 1; *FUR1*: Uracil phosphoribosyltransferase; *GanA*: G protein alpha subunit; *HapE*: CCAAT transcription factor; LOH, los of heterozygosity; *MRR1*: Multidrug resistance regulator 1; NA, not available; SNP, single nucleotide protein; *TAC1*: Transcriptional activator of *CDR* genes; *UPC2*: Sterol uptake control protein 2; SRA: Sequence Read Archived; ENA: European Nucleotide Archive.

Given that genome comparisons yield in most cases a large number of SNPs and/or indels, a major challenge is to identify the nucleotide alterations associated with drug resistance. For example, in a study in which 17 related sequential *C*. *albicans* isolates with increasing azole resistance (over a 23-month period) were analysed, 4,610 nonsynonymous SNPs accumulated compared to the initial susceptible isolate [30]. Without expert eyes, it is a daunting task to deduce SNPs associated with drug resistance among other SNPs resulting from host adaptations or simply genetic hitchhiking. Comparisons with genes known to be involved in azole resistance is the approach of choice when attempting to detect SNPs relevant for drug resistance. Alternatively, traditional genetics may be helpful in this task: the work of Losada and colleagues [31] showed that it is possible to cross A. *fumigatus* in vitro to generate drug-resistant isolates with isogenic susceptible isolates of opposite mating type and then to select resulting drug resistant progenies for genome analysis. Therefore, the number of SNPs not relevant for drug resistance is reduced, speeding up the discovery of drug resistance associated SNPs. However, there are a number of genome studies in which the divergence between susceptible and resistant isolates is not as extensive, which facilitates the detection of SNPs associated with drug resistance. For example, Branco and colleagues [32] obtained two related *C*. *parapsilosis* isolates, one of which was azole-resistant, and identified two heterozygous SNPs and one homozygous SNP, the latter in a gene encoding the C-5 sterol desaturase (*ERG3*). The resulting nonsynonymous mutation yielded a nonfunctional Erg3 protein, which triggered azole resistance. Of note is that the azole-resistant isolate was generated in vitro thus shortcutting the accumulation of unrelated (i.e., host adaptation) mutations in the genome. On the other hand, the study published by Vale-Silva and colleagues [29] used two clinical *C*. *glabrata* isolates, one of which developed azole resistance in a patient within a 50-day time lapse. The azole-resistant isolate accumulated only 17 nonsynonymous mutations, among which one was in the regulator *CgPDR1* known to mediate azole resistance. Very recently, comparative genome analysis helped to reveal an unexpected isolate diversity in *C*. *lusitaniae* isolates recovered from a single cystic fibrosis patient: the genomes of 20 different azole-resistant *C*. *lusitaniae* isolates from this patient revealed, among identified SNPs (24–131 between any two isolates) and indels (76–179 between any two isolates), accumulation of several distinct nonsynonymous SNPs in the transcription factor *MRR1*. This transcription factor is known to mediate azole resistance by upregulation of an efflux transporter [2], and therefore, the identified *MRR1* SNPs were likely to explain the occurrence of azole resistance in this species [33].

## Further steps

Other genome-wide analyses can be envisaged to correlate genotypes with phenotypes. One of them is the use of expression quantitative trait loci (eQTLs). eQTLs are naturally occurring genetic polymorphisms controlling gene expression levels; these analyses have proved to be a powerful approach for providing comprehensive data to interpret the link between genome variations and specific phenotypes [34]. This relatively novel approach has not yet been used in fungi-oriented studies; however, now that population-wide studies at the genome-scale are becoming popular, it promises to lead to interesting and novel findings.

In any of the discussed cases here, evidence is required to show the causal link between drug resistance and the candidate SNPs and/or indels identified by genome-wide studies. This supporting step has not been systematically addressed in the studies listed in Table 1. Nowadays, with the emerging technologies of gene editing (CRISPR [clustered regularly interspaced short palindromic repeats]-Cas9 [CRISPR-associated 9]), this task can be undertaken in several important fungal pathogens [35].

It is likely that in the future, more genome-based studies will emerge from fungal population analysis. Genome comparisons with known SNPs involved in drug resistance will still be the priority approach. Such comparisons may be facilitated by the creation of database resources, in which all known drug resistance genes and their allelic variants are deposited. This idea is currently attempted [36]; however, it should be strengthened by a strong commitment by the scientific community.
